# Emergence of porcine epidemic diarrhea virus in southern Germany

**DOI:** 10.1186/s12917-015-0454-1

**Published:** 2015-07-02

**Authors:** Julia Stadler, Susanne Zoels, Robert Fux, Dennis Hanke, Anne Pohlmann, Sandra Blome, Herbert Weissenböck, Christiane Weissenbacher-Lang, Mathias Ritzmann, Andrea Ladinig

**Affiliations:** Clinic for Swine at the Centre for Clinical Veterinary Medicine, Ludwig-Maximilians University, Oberschleissheim, Germany; Institute for Infectious Diseases and Zoonosis at the Centre for Clinical Veterinary Medicine, Ludwig-Maximilians University, Munich, Germany; Friedrich-Loeffler-Institut, Institute of Diagnostic Virology, Greifswald-Insel Riems, Germany; Institute of Pathology and Forensic Veterinary Medicine, University of Veterinary Medicine Vienna, Vienna, Austria; University Clinic for Swine, University of Veterinary Medicine Vienna, Vienna, Austria

**Keywords:** Porcine epidemic diarrhea virus, Southern Germany, Phylogenetic analysis, S INDEL PEDV

## Abstract

**Background:**

Over the last years, porcine epidemic diarrhea virus (PEDV) has caused devastating enteric diseases in the US and several countries in Asia, while outbreaks in Europe have only been reported sporadically since the 1980s. At present, only insufficient information is available on currently circulating PEDV strains in Europe and their impact on the European swine industry. In this case report, we present epidemic outbreaks of porcine epidemic diarrhea in three farms in South-Western Germany.

**Case presentation:**

Epidemic outbreaks of diarrhea affecting pigs of all age groups were reported in three farms, one fattening farm and two piglet producing farms, in South-Western Germany between May and November 2014. In the fattening farm yellowish, watery diarrhea without evidence of mucus or blood was associated with a massive reduction of feed consumption. Severity of clinical signs and mortality in young suckling pigs varied significantly between the two affected sow farms. While mortality in suckling piglets reached almost 70 % in one sow herd, no increase in suckling piglet mortality was observed in the second sow farm. In all three cases, PEDV was confirmed in feces and small intestines by RT-qPCR. Phylogenetic analyses based on full-length PEDV genomes revealed high identity among strains from all three herds. Moreover, the German strains showed very high nucleotide identity (99.4 %) with a variant of PEDV (OH851) that was isolated in the United States in January 2014. This strain with insertions and deletions in the S-gene (so called INDEL strains) was reported to show lower virulence. Slightly lower identities were found with other strains from the US and Asia.

**Conclusion:**

Phylogenetic information on the distribution of PEDV strains in Europe is severely lacking. In this case report we demonstrate that acute outbreaks of PEDV occurred in southern Germany in 2014. Current strains were clearly different from isolates found in the 1980s and were closely related to a PEDV variant found in the US in 2014. Moreover, the present case report indicates that variant strains of PEDV, containing insertions and deletions in the S gene, which were reported to be of lower virulence, might be able to cause high mortality in suckling piglets.

## Background

Porcine epidemic diarrhea virus (PEDV), an enveloped, single-stranded, positive-sense RNA virus, was identified as the causative agent of porcine epidemic diarrhea (PED) in 1978 [1]. It was subsequently placed into the order *Nidovirales,* the subfamily *Coronavirinae*, the family *Coronaviridae*, and the genus *Alphacoronavirus* [2]. PED was first described in the United Kingdom in 1971 [3]. While several outbreaks were reported across Europe in the 1970s, only sporadic outbreaks occurred in recent years [4, 5]. In contrast, PED has emerged as a devastating enteric disease causing severe economic losses in other major swine-producing countries, notably the United States and Asia, within the last years [6, 7]. Since the emergence of PEDV in the United States in April 2013, the disease has rapidly spread throughout the country, inducing explosive outbreaks of diarrhea with high mortality among infected suckling piglets [6, 8, 9]. Phylogenetic analysis of the original highly virulent US PEDV strain from April 2013 revealed high nucleotide identity to strains circulating in China in 2012 [6].

In January 2014, a new variant strain of PEDV (OH 851), containing insertions and deletions in the S gene (S INDEL), was identified in Ohio causing mild clinical signs and lower mortality rates in suckling piglets compared to other currently circulating PEDV strains in the US [10]. At present, both S INDEL and highly virulent non S INDEL strains are co-circulating in the US, though S INDEL strains are less frequently diagnosed [11] .

Fairly little information on the current situation on PED is available from European countries. Recently, several outbreaks of acute PED occurred in Germany. In this case report we describe acute outbreaks of PED in one fattening farm and two sow farms in South-Western Germany between May and November 2014.

## Case presentation

### Clinical presentation

#### Farm A (fattening farm)

In May 2014, an epidemic of diarrhea was reported in a 1200 head fattening farm located in an area with low pig density in Southern Germany. Two buildings separated by a distance of about 20 m are run in a continuous flow production, i.e. different age groups are housed in one barn. The pens are subdivided into an outdoor area with partially slatted floor and an indoor area equipped with concrete solid floor. On May 23, 2014, a new batch of 320 feeder pigs with an average weight of 30 kg was introduced into the fattening farm. The piglets had been purchased from a 14,000 head nursery facility in Northern Germany, which was single-sourced from a 5000 sow farrow to wean farm located in the Netherlands. On May 25, 2014 (two days after placement), the newly introduced pigs showed yellowish to greyish, watery diarrhea. According to the farmer, approximately 50 % of pigs from the youngest age group were affected. Feces contained undigested feed components but no mucus or blood. Vomitus was apparent in individual pigs. By the following day, all age groups within the same barn were affected. As of May 28, 2014, the disease had spread to the second barn. Within five to seven days after placement of the new group of feeder pigs, 95 % of all growing and finishing pigs developed diarrhea accompanied by anorexia and lethargy. The affected pigs showed varying degrees of weight loss and dehydration. Within the first ten days after the onset of clinical signs 20 pigs had died; 80 % of them originated from the youngest age group. Clinical signs subsided within three weeks in the older age groups, but recurrent diarrhea persisted in the youngest age group for up to 15 weeks. At the peak of clinical signs, five days after the onset of first clinical signs, feed consumption severely declined. Feed consumption returned to normal within six days in the older age groups but was compromised in the youngest age groups for several weeks. During the outbreak of PED an overall mortality rate of 4.5 %, an average daily gain (ADG) of 600 g and a prolonged fattening period of 30 days were documented. Prior to the outbreak, performance data accounted for 750 g ADG and 2 % mortality.

#### Farms B and C (sow farms)

The two sow farms are located in a pig dense area in South-Western Germany. In September 2014, farm B, which is producing piglets with 280 sows in a three week batch farrowing interval, was experiencing diarrhea in all age groups. First clinical signs were characterized by anorexia in lactating sows and diarrhea of the corresponding three week old piglets. Pasty diarrhea progressed to watery greyish feces with variable degrees of dehydration, affecting about 70 % of piglets from one farrowing batch. Two days after the first onset of diarrhea in suckling piglets, up to 100 % of the sows in the farrowing unit were affected by watery diarrhea. Within one week after the onset of first clinical signs, diarrhea had spread to all age groups of pigs present at the farm including boars and nursery pigs. During the outbreak, mortality ranged from 5.5 % in suckling piglets to 8.8 % in nursery pigs. Clinical signs were present for approximately four weeks and performance data returned to normal within six weeks after the outbreak of PED.

**Fig. 1:**
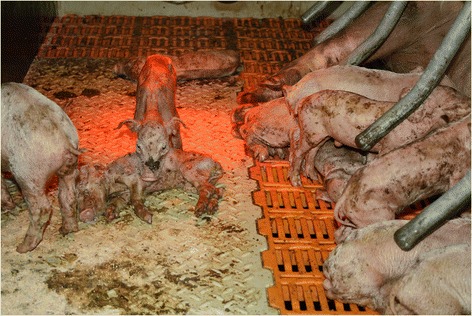
Suckling piglets with severe diarrhea and dehydration. Seven-day-old piglets from a severly affected litter from farm C. Piglets, covered with feces, showing yellowish, pasty to watery diarrhea and varying degrees of dehydration

Clinical signs in farm C, a piglet producing farm with about 290 sows and a nursery unit for 700 piglets, started on October 30/November 1, 2014. The group of sows weaned on October 30 showed severely reduced feed intake for the first seven to eight days after weaning and had yellowish, watery diarrhea. More than 95 % of sows from that group were affected. Two to three days after the clinical signs had been observed in the breeding area, diarrhea and reduced to absent feed intake occurred in the farrowing unit in sows which were nursing piglets of about 2.5 weeks of age. Suckling piglets started with mild pasty, yellowish diarrhea but were not severely affected and only three piglets died before weaning. The next group of sows farrowed between November 7 and 9. Piglets from those sows were acutely affected and started to show yellowish, pasty to watery diarrhea about two days after birth. First piglet losses occurred on November 10. In total, 67.6 % of piglets from this farrowing group died or had to be euthanized before weaning. Diarrheic, gaunt and dehydrated piglets, covered with feces (Fig. 1), were found in 30 out of 35 (86 %) litters from this farrowing group. In addition to signs in piglets, sows had watery diarrhea and severely reduced feed intake. Pasty to watery diarrhea also occurred in the gestation unit where sows are housed in small groups of six to eight animals on slatted floor. Clinical signs in the nursery mainly consisted of severely reduced feed intake and diarrhea in individual pigs. Mortality in the nursery reached up to 7.1 % during the outbreak. Clinical signs subsided within four weeks after the onset of diarrhea. No diarrhea was observed in suckling piglets of the consecutive farrowing group born around November 26.

### Laboratory findings

**Fig. 2:**
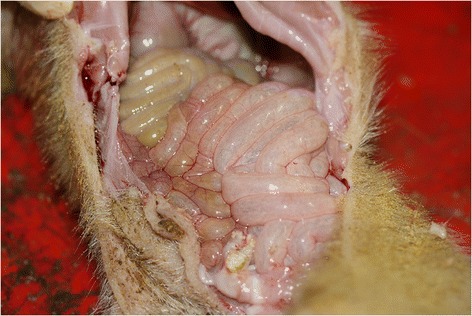
Macroscopic changes in the small intestines from a suckling piglet of farm C. Gross lesions are characterized by thin and transparent intestinal walls and accumulation of large amounts of yellowish fluid in the intestinal lumen

**Fig. 3:**
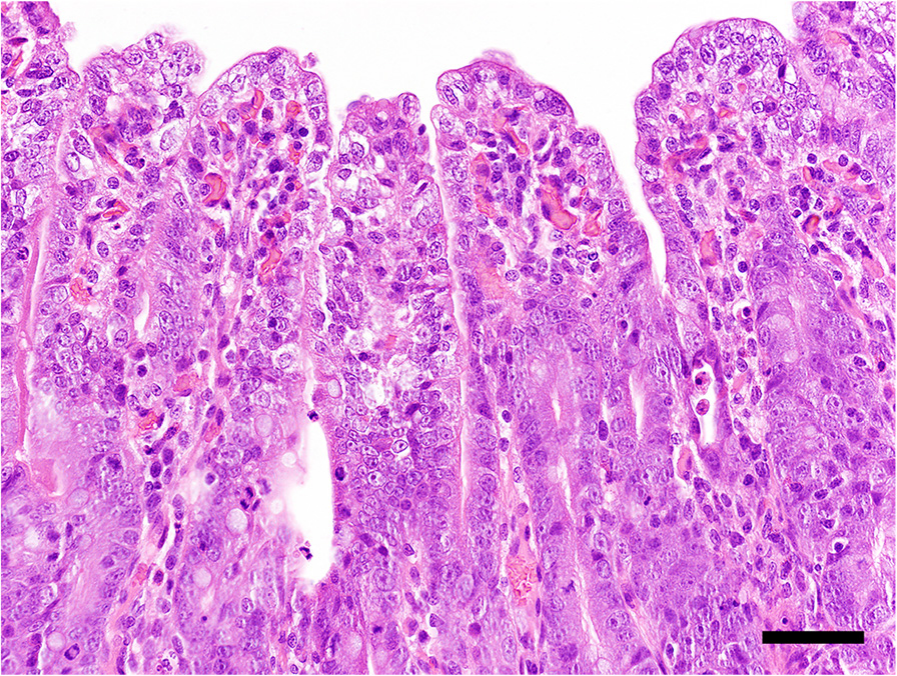
Histological changes in the small intestines from a suckling piglet of farm C. There are shortened, blunted and partly fused villi. The cytoplasm of the villous enterocytes is markedly vacuolated. Haematoxylin and eosin staining, Bar = 80 μm

**Fig. 4:**
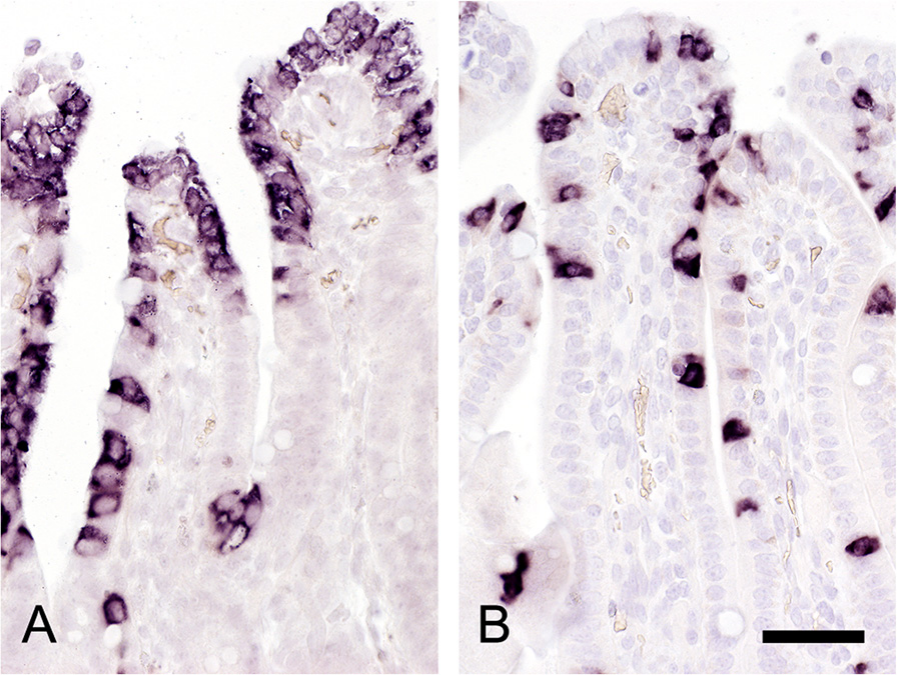
Localization of viral nucleic acid in enterocytes by *in situ* hybridization. **a** The cytoplasm of the majority of villous enterocytes is positively labelled in a case with +++ score. **b** Only few, scattered enterocytes are positive in a case with + score. Bar = 80 μm

Severely affected animals from all three farms (Farm A: 3 feeder pigs, Farm B: 4 suckling piglets, Farm C: 25 suckling piglets) were submitted for pathological investigations. Gross lesions were limited to the intestines and characterized by distended, thin and transparent intestinal walls, mainly observed in the small intestines but partially also in the colon region (Fig. 2). The intestinal lumen contained yellow, watery and frothy fluid. In individual pigs intestinal contents were completely absent. Tissue samples from small intestines were fixed in 10 % neutral buffered formalin and embedded in paraffin wax. Tissue sections were stained with hematoxylin and eosin (H&E) for microscopic examination by standard methods. Histology was characterized by atrophic enteritis, which included shortening, blunting and fusion of the villi, occasionally with vacuolation and exfoliation of enterocytes (Fig. 3). There were individually differing grades of the lesions. In farm C a more detailed evaluation of histological lesions and an additional localization of viral nucleic acid within lesions was performed due to the availability of higher numbers of severely affected piglets. In four of the 25 investigated piglets lesions were categorized as severe, in nine piglets as moderate and in 12 piglets as mild (Table 1). A chromogenic *in situ* hybridization (ISH) procedure was developed for semi-quantitative analysis of viral nucleic acid present within enterocytes. An oligonucleotide probe with the potential to hybridize with all representatives of PEDV was designed. Probe design was based on extensive homology studies on available nucleocapsid gene sequences from GenBank using the software Sci Ed Central for Windows 95 (Scientific & Educational Software, Cary, NC, USA). The resulting probe sequence was: 5’-GCATCCTTGACAGCAGCCACCAGATCATCGCGTGAT-3’. The probe sequence was submitted to Basic Local Alignment Search Tool (BLAST) [12] to search against GenBank sequences and to exclude unintentional cross-reactivity with other organisms. The ISH procedure followed a previously published protocol [13]. The probe concentration was 35 ng/μl. As negative controls paraffin wax-embedded small intestinal tissue samples of a healthy piglet and of a piglet experimentally infected with transmissible gastroenteritis virus (TGEV), an unrelated alphacoronavirus, were used. The proportion of positive enterocytes was assessed semiquantitatively using the score +++ for abundant (between 75 % and 100 % of enterocytes positive), ++ for moderate (between 25 % to 75 % of enterocytes positive) and + for few (less than 25 % of enterocytes positive) signals. By ISH, viral signals were found exclusively in the cytoplasm of enterocytes (Fig. 4). The vast majority of infected cells was present in the villi but occasionally there were positive crypt epithelia too. In 9 piglets necropsied from farm C there were abundant ISH signals (+++) and in 8 cases each, moderate (++) or few (+) signals were detected. The amount of positive cells did not exactly correspond with the histological scores (Table 1).

**Table 1:** Histological scores and in situ hybridization results of 25 piglets from farm C

Piglet No.	Histological score	ISH score
1	+	+++
2	+	+
3	++	++
4	+	++
5	++	+
6	+++	+++
7	++	+++
8	++	+++
9	++	+++
10	+	+
11	+	+
12	+	+
13	++	+
14	+	++
15	+	+
16	++	++
17	++	++
18	+++	+++
19	+++	++
20	+	+++
21	+	+++
22	+++	+++
23	+	++
24	+	+
25	++	+++

Electron microscopy was performed on fecal and tissue samples at the Institute for Infectious Diseases and Zoonosis, Ludwig-Maximilians University, Munich. Coronavirus-like particles were identified on the basis of their typical morphological characteristics.

For PEDV diagnosis pooled fecal samples (Farm A: n = 5 ; Farm B: n = 8; Farm C: n = 4) and small intestinal tissue samples (Farm A: n = 3; Farm B: n = 4; Farm C: n = 3) were investigated at the Institute for Infectious Diseases and Zoonosis, Ludwig-Maximilians University, Munich using two independent RT-qPCR systems targeting the PEDV N- and S-genes, respectively [14, 15]. The presence of PEDV-specific nucleotide sequences was confirmed in fecal and tissue samples of all three farms by RT-qPCR. All fecal samples were negative for TGEV, determined by RT-PCR [15]. For differential diagnosis fecal samples from all three farms were investigated for the presence of porcine Rotavirus, porcine Deltacoronavirus and porcine Norovirus by virus-specific RT-PCRs as described previously [16–18]. Additionally, fecal samples from both piglet producing farms were examined for porcine Sapovirus by RT-PCR [19]. All samples were negative for the aforementioned enteric viral pathogens. To further investigate concomitant infections, bacteriological investigations were performed on fecal samples from diarrheic pigs from all three farms. The following bacteria were isolated: *Salmonella derby* and *E. coli* (positive for F18 and Shigatoxin 2e) in farm A, ST-II and ST-IP producing *E. coli* in farm B, and *E. coli* without major virulence factors (including LT-1, ST-IP, ST-II, Shigatoxin 2e, F4, F5, F6, F18, F41 and Intimin) and *Clostridium perfringens* type A negative for the enterotoxin gene and the beta 2 toxin gene in farm C.

Subsequently, in farm A and farm B five pooled fecal samples, originating from different age groups, were tested for PEDV by RT-qPCR on a biweekly basis to monitor the on-farm infection dynamics. PEDV-specific nucleotide sequences could be detected in all fecal samples obtained from farm A up to 15 weeks after the onset of first clinical signs. Fecal samples from farm B collected four, six and eight weeks after the initial occurrence of diarrhea were negative for PEDV RNA. Nine weeks after the onset of clinical signs in farm B, diarrhea recurred in the group of pigs which was first affected by clinical signs. PEDV nucleic acid could be detected in fecal samples collected from affected pigs.

**Fig. 5:**
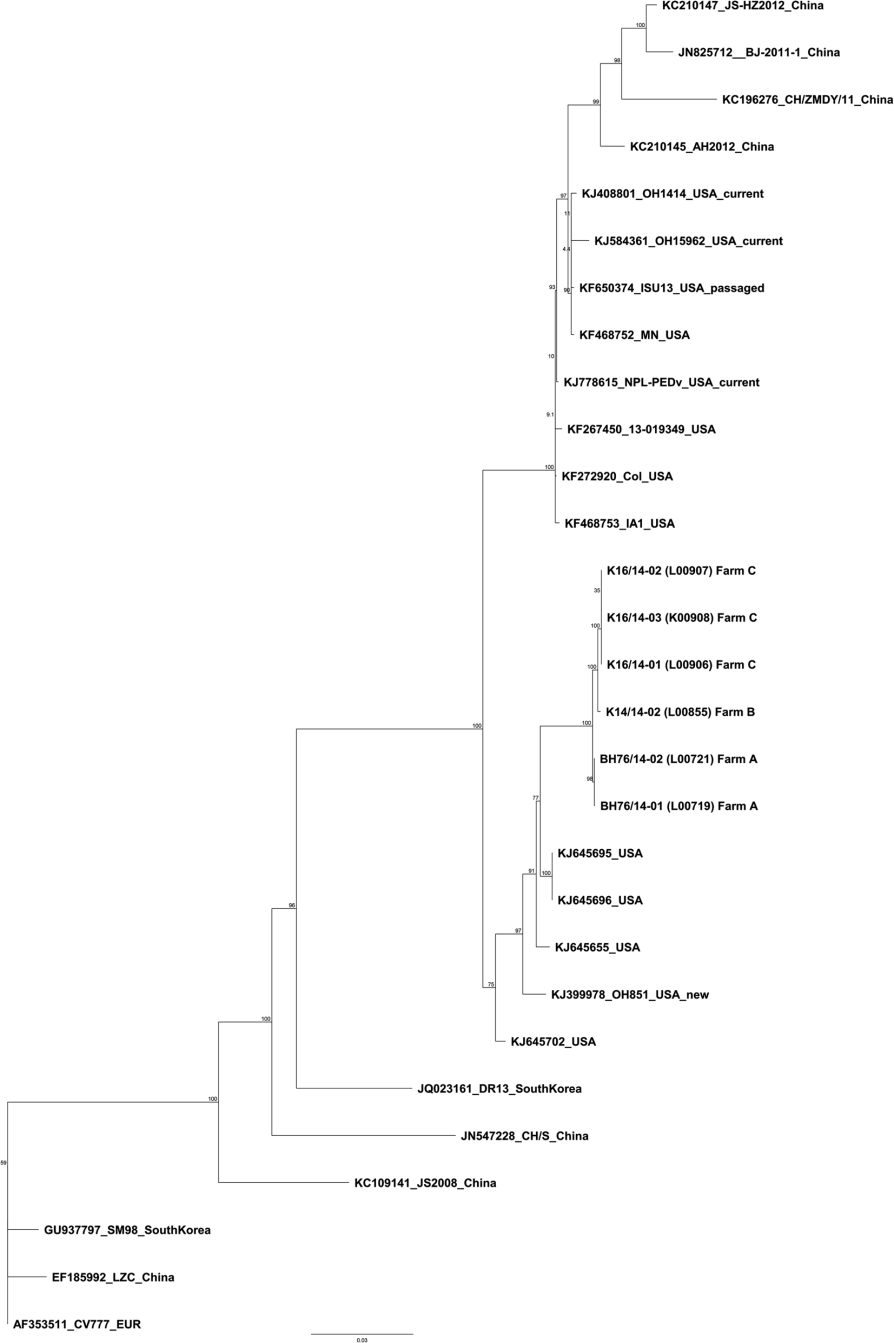
Phylogenetic tree of current German PEDV strains compared with strains from the United States and China. Full genome sequences of German PEDV strains were aligned using MAFFT, and phylogenetic analyses were carried out using the maximum-likelihood method PhyML [22] with a GTR substitution model and tree reconstruction supported by 1000 bootstrapping replicates. All tools implemented in Geneious (vs. 8.0.5, Biomatters [30]). Strain derived from farm A are found as BH76/14, from farm B as K14/14, and farm C as K16/14

Selected positive fecal samples from all three farms were submitted to the Friedrich-Loeffler-Institute, Isle of Riems, Germany for full genome sequencing and virus isolation. PEDV was successfully isolated from one fecal sample of farm A following the protocol published by Oka *et al.* [20] with slight modifications. Supplemental investigations are ongoing. Full-length genomes were determined using next-generation sequencing as previously described [21]. Nucleotide sequence alignment analysis of the full-length genomes revealed up to 98.7 % nucleotide identities with currently circulating highly virulent US strains and strains from China. Highest similarity existed to the so-called S INDEL strains described in the US [10]. The German PEDV strains shared approx. 99.4 % identity with those strains (OH 851 as an INDEL prototype strain). Much lower overall similarity was found between the current German S INDEL strains and the European isolate CV777 from the late 1970s. Phylogenetic trees were constructed for complete genomes and Spike protein sequences using PhyML [22] in the Geneious software suite with a GTR substitution model and supported by 1000 bootstrapping replicates. Since the phylogenetic tree based on the Spike gene did not add more information than obtained from the full-length tree we decided not to include it in the manuscript. All German isolates clustered together and with OH 851 (Fig. 5). Based on the data obtained from all three farms no recombination events were observed.

## Discussion

PEDV emerged and spread in the 1970s and 1980s in several European countries. The disease was characterized by watery diarrhea and affected all age classes of animals. However, the impact of PED on the European swine industry has remarkably decreased after the 1980s. Over the last ten years only sporadic outbreaks of PED combined with low mortality rates have been reported [4, 5, 23], thus requiring no active monitoring of PEDV in Europe. Moreover, serological data on the current prevalence of PEDV in Europe are scarce and further restricted by the limited sensitivity and specificity of the hitherto available diagnostic tests [24].

In contrast, PED has emerged as a devastating disease causing substantial economic losses in Asia and North America over the last years [8, 9]. Since the first introduction into the US in April 2013, PED has spread to 32 US states [25] and has also been introduced into Canada [26]. Soon after the emergence of PED in the US, sequence analyses revealed high nucleotide identity between North American and current Asian PEDV strains suggesting a common ancestor for PEDV in both continents [27, 28]. At the beginning of 2014, new variants of PEDV showing distinct genetic patterns in the S gene, i.e. deletions, an insertion and several mutations in the first 1170 nucleotides of the S1 region, were found in the US [10]. Those PEDV strains, also called S INDEL PEDV, were reported to be of lower virulence inducing mild to absent clinical signs and low mortality in suckling piglets [10]. PEDV strains described in this case report were most closely related to S INDEL PEDV. Similar strains were found in North-Western Germany, where outbreaks of PED characterized by high morbidity but no mortality occurred in three fattening farms in spring 2014 [29]. In contrast to the short duration of diarrhea observed in the North Western German fattening farms, diarrhea in the fattening farm described in this case report (Farm A) lasted up to 15 weeks after the initial outbreak and was associated with a massive decline in ADG and an increased mortality rate. This might be due to management and housing conditions in farm B, but could also be related to co-infections complicating the clinical disease.

Besides PED outbreaks in German fattening farms, the occurrence of PED in two sow farms in Southern Germany has unsettled German pig farmers. The severity of clinical signs observed in the two affected sow farms differed significantly, although sequence analyses revealed that strains found in both farms showed 99.9 % nucleotide identity based on full genome sequencing with 12 different nucleotides found in the genome, four of which were located in the spike protein coding sequence. The manifestation of the disease might vary between herds due to the level of immunity within the population, potential infectious co-agents as well as management and biosecurity reasons. Since no diarrhea occurred in suckling piglets in farm C prior to and after the acute outbreak of PED, we concluded that co-infections played a minor role in this farm, which is supported by the fact that no relevant enteric viral pathogens were found and bacteria isolated from diarrheic piglets were negative for known genes of major virulence factors.

## Conclusion

In conclusion, the present case report reveals that PEDV strains similar to S INDEL PEDV are currently circulating in different farm types in Southern Germany. Severe clinical signs and high mortality rates in suckling piglets observed in one farm might indicate that the potential of virulence can vary between S INDEL PEDV strains. However, experimental studies to confirm the potential of high virulence of S INDEL PEDV are lacking to date.

Due to insufficient data on phylogenetic analysis of PEDV strains in Europe we cannot clarify if the reported strains were recently introduced from the US or have been circulating in Europe since the 1970s. Further molecular epidemiologic research is therefore needed to find temporal and geographic evidence for the exact origin and evolution of the recent PEDV strains in Germany.

## Abbreviations

PEDV: Porcine epidemic diarrhea virus
PED: Porcine epidemic diarrhea
ADG: average daily gain
BLAST: Basic Local Alignment Search Tool
RT-qPCR: quantitative reverse transcriptase polymerase chain reaction
*E.coli*: *Escherichia coli*
LT-I: *Escherichia coli* heat-labile enterotoxin I
ST-IP: *Escherichia coli* heat-stable enterotoxin IP
ST-II: *Escherichia coli* heat-stable enterotoxin II
S INDEL: PEDV strains containing distinct insertions and deletions in the S gene
